# The Hummingbird Project: A Positive Psychology Intervention for Secondary School Students

**DOI:** 10.3389/fpsyg.2020.02012

**Published:** 2020-08-07

**Authors:** Ian Andrew Platt, Chathurika Kannangara, Michelle Tytherleigh, Jerome Carson

**Affiliations:** ^1^School of Education and Psychology, University of Bolton, Bolton, United Kingdom; ^2^School of Psychology, University of Chester, Chester, United Kingdom

**Keywords:** school, happiness, well-being, Positive Psychology, child, adolescent, universal, preventative

## Abstract

Mental health in schools has attracted a lot of attention in recent years. Positive Psychology Interventions (PPIs) in secondary schools have been shown to improve mental health outcomes for students. Previous PPIs have tended to be delivered by trained Psychology specialists or have tended to focus on a single aspect of Positive Psychology such as Mindfulness. The current study involved 2 phases. Phase 1 was a pilot PPI, delivered by current university students in Psychology, which educated secondary school students (*N* = 90) in a variety of Positive Psychology concepts. Phase 2 involved delivering the PPI to secondary school students (*N* = 1,054). This PPI, the Hummingbird Project, led to improvements in student well-being, as measured by the World Health Organization Well-Being Index (WHO-5). The intervention also led to improvements in student resilience, as measured by the Bolton Uni-Stride Scale (BUSS), and hope, as measured by the Children’s Hope Scale (CHS). Results are discussed in the context of their implications for the future of psychological intervention in secondary school settings.

## Introduction

Children spend significantly more time in school compared to any other formal institution during their lives (Rutter et al., 1979), though school hours have changed very little in the last 40 years (Symonds and Hagell, 2011). Consequently, schools play a major role in children’s development, including relationships, cognitive development, social skills, academic attainment, emotional, and behavioral control (Fazel et al., 2014). As such schools have increasingly been the prime target of interventions designed to improve young peoples’ mental health (Wyn et al., 2000). It was argued by Campion et al. (2012) that mental health and mental disorder are distinct. Absence of one does not imply the presence of the other. However, prevention of mental disorder is closely associated and can be considered as a product of promoting mental health and resilience. It is considered that health, social and economic benefits are products of positive mental health (Lyubomirsky et al., 2005; Keyes, 2007). The following factors are associated with positive mental health; improved educational attainment, greater productivity, reduced mortality, increased social interaction and engagement, reduced risk of suicide and mental illness, reduced likelihood of risk-taking behavior, and increased levels of resilience to adversity (Campion et al., 2012).

According to World Health Organization statistics (WHO, 2008), in the United Kingdom, mental disorder accounts for 22.8% of the total health burden, cancer for 15.9% and cardiovascular disease 16.2%. These findings can be seen as alarming and could impact on the UK’s future social and economic stability. It has been shown that 50% of lifetime mental illness starts by age 14 and 75% by the mid-20s (Kessler et al., 2007). As such, early interventions for the prevention of mental disorder during childhood and adolescence could prove helpful. In the UK more than one in 10 children and young people are reported to have a mental health disorder at any one time (Green et al., 2005). Mental health costs the UK economy £70–100 billion each year, or 4.5% of its Gross Domestic Product (GDP) (Goldie et al., 2016). A longitudinal study analyzed the data of 17,634 children from England, Scotland and Wales, and found associations between childhood psychological problems and the ability of affected children to work and earn as adults (Goodman et al., 2011).

The British Office for National Statistics has shown that the North West of England contains five of the 10 most deprived local authorities in England (Gill, 2015). The proportion of the population in England who are in the most deprived group varies from a low of 7.3% in the South East to a high of 32.8% in the North West (Newton et al., 2015). Deprivation has been linked to a variety of negative outcomes in both physical and mental health. It is reported that the UK is facing a significant level of mental health inequality despite major government policy initiatives (Grey et al., 2013). In parallel, there has been a reduction in National Health Service (NHS) expenditure on specialist mental health care since 2008 (Docherty and Thornicroft, 2015). Some local authorities have experienced reductions in spending of up to 32%. The report claims that this spending reduction has contributed to a 48% decrease in the number of people with mental illnesses receiving appropriate care. This reduction is also present in Child and Adolescent Mental Health Services (CAMHS). A survey of members of the Faculty of Child and Adolescent Psychiatrists into experiences of admitting young people to inpatient units (Hindley, 2014) showed 77% of respondents had experienced difficulties when accessing admissions to inpatient beds. Some 79.1% reported experiencing safeguarding concerns or incidents whilst waiting for a bed and 76% reported that a lack of beds had led to young people with unacceptably high-risk profiles being managed in the community. Finally, 61.9% reported that they had experience of young people being held in inappropriate settings. The mental health outcomes of children across the country will suffer due to issues such as these.

Recent research highlights an unprecedented increase in childhood and adolescent depression among Western countries. At any point of time, approximately 2% of children aged 11–15 and 11% of youth aged 16–24 are suffering from a major depressive disorder in the UK (Green et al., 2005). Boniwell and Ryan (2012) argue that wealthier countries appear to invest relatively little in protection for the safety of young people. In 2007, a UNICEF report presented an overview of child well-being in rich countries, where the UK was in the bottom third of the list of 21 countries. The UK did in fact come last under the dimension of subjective well-being, where children ranked their opinion of their health, interest in schools, and subjective view of their personal well-being. This alone shows the need to intervene in Britain’s schools.

Over the last 20 years, Positive Psychology (PP) has developed a science of well-being (Seligman, 2002) and a growing body of empirical evidence supports Positive Psychology Interventions (PPIs). The first manualized treatment interventions are now being produced (Slade et al., 2017; Rashid and Seligman, 2018). There are core pillars behind the PP-based well-being approaches such as the science of character strengths, health, achievement, and a range of other positive outcomes (Niemiec, 2013). Well-being interventions can have a preventive effect on depression, anxiety, and other mental health disorders.

A systematic review and a meta-analysis conducted by Werner-Seidler et al. (2017) included 81 studies conducted in schools where students had undergone some sort of depression and anxiety prevention program. It identified three UK based studies whereas there were 27 studies from America and 20 studies from Australia. The UK needs to expand its provision. The need for a scientific basis for these interventions is considered critical. The assessment of the intervention should be carefully designed to yield accurate results. Classroom-based intervention studies are mainly either universal or targeted. There is evidence from previously effective studies to demonstrate that a combination of universal and targeted interventions may have a positive impact. The BRIDGE program links mental health and education in urban schools. Conclusions from a randomized controlled study (Fazel et al., 2014), showed the program was effective at the classroom and individual level and resulted in positive outcomes. The program was developed and designed in New York and targeted at elementary level schools (Cappella et al., 2012). There is a need for an empirically tested intervention in the UK school context. An obstacle to the implementation of evidence-based interventions in school settings is “poor engagement” of all necessary parties such as school staff, counselors, and support staff. There are also other challenges such as individual factors (e.g., stigma, risk factors, parental issues), community-related factors, and system-related factors such as access to funding, waiting times, and availability of training (Fazel et al., 2014).

Building happiness into the school curriculum can be a successful approach to intervening in student mental health (Boniwell et al., 2016). This can be achieved by having PPIs that run as a year-round addition to the timetable (Shoshani and Steinmetz, 2014). Approaches such as these require support on a number of levels to embed PP into teacher training, school leadership training, and into the system-wide educational culture (Waters, 2011). The costs involved could be prohibitive. Shorter-term PPIs that are not embedded in the school curriculum have shown mixed results (Suldo et al., 2014). There is an insufficient evidence base to support the widespread adoption of PPIs in schools (Dawood, 2013). However, there remains cause for optimism as some PPIs have been shown to effectively increase students’ motivation to study and enhance their academic performance (Muro et al., 2018). A review of Brief PPIs has shown that they can be successfully implemented and demonstrate a diverse range of benefits to students in terms of their learning and their well-being (Shankland and Rosset, 2017). However, these brief PPIs typically focus on a single aspect of PP such as gratitude (Froh et al., 2009), character strengths (Quinlan et al., 2015), or mindfulness (Sapthiang et al., 2019). One intervention that has been shown to be effective in improving mental health outcomes, which operates by improving social and emotional skills, is KidsMatter (Littlefield et al., 2017). KidsMatter has also been shown to be favorable to pupils. Insufficient consideration of children’s views can result in a disparity between what young people say about their lives and their needs and what problems are targeted by an intervention (Kidger et al., 2009). This might then result in pupils having negative views of existing support. If children do not view an intervention as being confidential, available to all, or sympathetic to their needs, this issue can be exacerbated. Stigma was one of the main concerns highlighted by the children who took part in this study. These children were wary of being seen or treated differently than their peers. Kidger et al. (2009) showed that this fear has the potential to create a significant barrier, preventing children from accessing a particular source of support. Findings of this type highlight the importance of ensuring that pupils are informed and have readily available information about what interventions and services are provided. It is recommended that pupils’ opinions of services are surveyed so that provision can be altered to better meet their needs.

Whole-school reviews of values, policies, and practices suggest that schools use separate initiatives alongside mainstream teaching to address mental well-being (Spratt et al., 2006). The report suggests that schools should employ specialist mental health workers to build a school climate that benefits all pupils. Embedding a holistic approach to mental health into standard teaching practice has been shown to improve outcomes for a range of age groups (White and Waters, 2015). Using this holistic approach and focusing less on mental health specialization could potentially help educators to build relationships with the children they teach such that they can identify and intervene in their well-being since, in this circumstance, it is the people who teach the children who intervene in their mental health. The qualities children and adolescents consider most important in mental health professionals (Farnfield and Kaszap, 1998) are general helpful qualities, counseling skills, an ethical stance, and providing helpful outcomes. What matters most to children is not the profession of the adult but that the adult possesses these qualities. However, unless it comes with a recommendation from a colleague whose opinion they respect, Head Teachers find it difficult to know whether a service being offered is of sufficient quality (NAHT/Place2Be, 2017).

Child and adolescent academic and mental health functioning can be promoted and improved by both mental health identification programs and prevention programs when delivered in schools (Levitt et al., 2007). Who will deliver these programs? In real terms, the Department for Education budget was cut by 7.4% between 2010 and 2016 (Crawford and Keynes, 2015). This lack of money for schools leads to staff feeling unable to deal with pupils’ mental health adequately. Studies have found that many teachers report their jobs as being very or extremely stressful (Nagel and Brown, 2003). While teachers in Norway report high job satisfaction, they also report suffering from severe stress and even exhaustion (Skaalvik and Skaalvik, 2015). Initial teacher training in mental health is varied at best. In many cases, it is non-existent (Byrne et al., 2015). Teachers require appropriate knowledge, skills, and attitudes if they are to recognize mental health problems (Whitley et al., 2013). Teachers also need to be aware of what steps need to be taken to integrate the affected pupils in activities in the classroom and to obtain for them the care they require. When children display both internalizing and externalizing of their problems, teachers are able to identify possible mental illness (Vieira et al., 2014). However, the same study demonstrated that this was not the case when children only showed internalizing behaviors. There is room for cautious optimism, as at least half of teachers involved in the study could identify problem cases and make the appropriate referral, with the correct training. Indeed, 60% of teachers who failed to identify typical adolescent behaviors at the beginning of the study were able to be trained in this skill. Training educators in order to improve their knowledge of, and attitudes to, mental illness has been highlighted as an important next step (Kutcher et al., 2013). Improving educator knowledge and attitudes is also highlighted as an important next step so that schools can provide effective mental health promotion and prevention. It may be more prudent to find low-cost interventions that can be delivered with minimal training.

Before intervening in the situation described above, it was decided that it would be helpful to scope the opinions of Head Teachers and Special Educational Needs Coordinators (SENCOs) regarding the main causes of mental ill-health amongst school-aged children in the North West of England. Scoping looked at the current situation in children’s mental health services, as well as mental health interventions in the region’s schools. This might clarify areas where intervention into children’s mental health was most needed and inform the direction of both future research and interventions.

Results from the scoping study showed educators considered deprivation as the main cause of mental ill-health in children and adolescents in the North West of England. This confirms a relationship between deprivation and a range of health outcomes. Family issues, including domestic violence and divorce, were more likely to be experienced by deprived children. These problems were seen as being exacerbated by deprivation, though they were not seen as being exclusive to deprived children. This is consistent with previous research demonstrating the relationship between deprivation, family dissolution, and academic performance (Downey, 1994; Pong and Ju, 2000). Scoping also showed that educators believed there was a skills gap when it came to dealing with mental health problems in children in the North West of England. Educators saw this skills gap as being exacerbated by a lack of money, staff, and training for school staff to provide for child and adolescent mental health. This shows that a unique resource burden is associated with attempting to embed mental health interventions in schools (Fazel et al., 2014). Educators in the North West of England felt underfunding meant children were being failed both at school and in specialist mental health services. These educators see specialist mental health services as failing to meet the needs of the pupils at their schools. They believed that waiting times were too long for such services to be of use to children. This is consistent with existing research (Frith, 2016).

### Theoretical Framework

Based on the issues discussed above, a new psycho-educational PPI, the Hummingbird Project, was created and piloted in two schools in Greater Manchester. The hope here was that an intervention could be created that would overcome the issues raised whilst incorporating the factors highlighted as being important when intervening in child and adolescent mental health in a school setting. The study used a theoretical approach grounded in PP. It was anticipated that teaching children and adolescents concepts from PP and allowing them to practice activities based on these concepts at home would lead to an improvement in well-being. The approach taken was empirically based. However, given the funding and time pressures in the educational setting discussed above, it was deemed impractical to perform a full randomized controlled trial. Therefore, a pragmatic approach using within-subject comparisons was necessary.

### Aim and Research Question

The aim of the current study was to deliver a short, evidence-based PPI in High Schools that could be replicated by school staff with minimal training. This would be achieved by recruiting schools in the North West of England to participate in the intervention and delivering the intervention, with the assistance of MSc and BSc Psychology students from two local universities. The PPI would then be evaluated using standardized measures, administered to participants in the first and last sessions. In this way it was hoped to answer the research question: Can a short, multi-component PPI be successfully delivered in Secondary Schools in the North West of England to improve well-being in pupils?

## Materials and Methods

The PPI involved 8 weekly 1 h sessions, taking place in the two schools during normal timetabled hours, delivered by MSc Psychology students. Session 1 involved an introduction to the program for students and teachers. Sessions 2–7 covered a range of concepts from Positive Psychology. These sessions covered happiness and well-being, grit and resilience, growth mindsets and character strengths, mindfulness, mental health problems and stigma, and hope and gratitude, respectively. The final session involved an overview and recap of the topics covered as well as advice for students on how to use the concepts discussed during the PPI in their everyday lives.

### Participants

The pilot PPI involved an opportunity sample of 90 students from two schools. Participants were recruited by emailing the Head Teachers and SENCOs of several schools in the region. Inclusion criteria were studying at a secondary school and a willingness to take part in the study. Participant ages ranged from 11 to 16 and 66% were female. Each student received a workbook in which they could record all of the work they did in sessions and at home, as well as any thoughts and feelings they had about the experience. They also received questionnaires as outcome measures.

### Instruments

Questionnaires included four standardized quantitative measures. These were:

(1) The World Health Organization-Five Well-Being Index (WHO-5) (Staehr, 1998), a five-item well-being scale on which items are measured on a six-point Likert scale (0 = at no time; 5 = all of the time). WHO-5 Cronbach’s Alpha = 0.84 and an example item is “I have felt cheerful and in good spirits.” The WHO-5 has been validated as a measure of well-being for adolescents (De Wit et al., 2007) using confirmatory factor analysis (*r* = 0.60, *p* < 0.01).
(2) Grit was measured using the Grit Scale (GRIT) (Duckworth et al., 2007), a 12-item scale on which items are measured on a five-point Likert scale (1 = not like me at all; 5 = very much like me). GRIT Cronbach’s Alpha = 0.77 and an example item is “Setbacks don’t discourage me.” Duckworth et al. (2007) demonstrated the validity of the GRIT scale using confirmatory factor analysis (chi-square (19, *n* = 1,554) = 188.52, *p* < 0.01). They also demonstrated 1 year test-retest stability for children and adolescents (*r* = 0.68, *p* < 0.01).
(3) The Bolton Uni-Stride Scale (BUSS) (Kannangara et al., 2020) was used to measure academic tenacity. The BUSS is a 12-item scale on which items are measured on a five-point Likert scale (1 = not like me at all; 5 = very much like me). Cronbach’s Alpha = 0.64 and a sample item is “I consider myself as persistent and hard-working.” Using a sample of 340 undergraduate students, Kannangara et al. (2020) demonstrated that the BUSS has good internal consistency (Cronbach’s alpha = 0.74) and 3-week test-retest reliability (Cronbach’s alpha = 0.70 for the Tenacity subscale and 0.77 for the Self-composure subscale.
(4) The Children’s Hope Scale (CHS) (Snyder et al., 1997), a measure of hope that uses six items on a six-point Likert scale (1 = none of the time; 6 = all of the time). CHS Cronbach’s Alpha = 0.91 and an example item is “I can think of many ways to get the things in life that are most important to me.” Snyder et al. (1997) demonstrated that the CHS has high internal consistency (Cronbach’s alpha = 0.72–0.86) and high temporal stability [*r*(359) = 0.17, *p* < 0.01].

### Data Collection Procedures

Outcome measures were administered to participants at the start of session 1 and the end of session 8 of the PPI. Participants were asked to be as honest as possible and informed of their right to withdraw from the study. They were informed that school grades would not be affected by participation in the study or by their answers to the questions. Upon completion of pre- and post-intervention questionnaires, questionnaires were coded as stated above, with negatively coded items being reverse scored. Only the GRIT and BUSS scales contained negatively coded items. An example of a negatively coded item from the GRIT scale is item 2, “New ideas and projects sometimes distract me from previous ones.” An example of a negatively coded item from the BUSS is item 4, “Sometimes I know that my actions are wrong, but I cannot stop myself.”

### Data Analysis Procedures

Data was analyzed using SPSS (version 25). Using this statistical program, descriptive statistics were generated and reliability analyses were conducted for all scales. Kolmogorov-Smirnov tests showed that data were normally distributed for all four scales; WHO-5 D(88) = 0.08, *p* = 0.20, GRIT D(31) = 0.13, *p* = 0.20, BUSS D(53) = 0.09, *p* = 0.20, CHS D(87) = 0.09, *p* = 0.16. Therefore, paired samples *t*-tests were conducted to compare scores on the four scales before and after participation in the PPI and to analyze the impact of the PPI.

## Results

Paired sample t-tests were performed. Table 1 shows descriptive statistics for these tests. The only measure on which a statistically significant change was found was the WHO-5, showing that participants’ levels of well-being had improved over the 8 weeks of the Hummingbird Project. Cohen’s d shows a small to medium effect size on this measure. Changes in the other measures used were shown not to be statistically significant. However, there were small positive improvements on all of the measures used.

**TABLE 1:** Descriptive statistics for pilot study.

Scale	n	Start mean	*SD*	End mean	*SD*	*t*	*p*	Cohen’s d
WHO-5	64	10.55	5.71	11.86	6.16	−2.71	0.01	0.34
Grit	29	37.07	6.98	37.14	7.18	−0.07	0.95	0.01
BUSS	32	33.50	6.38	34.50	7.25	−1.42	0.17	0.25
CHS	63	20.06	7.76	21.13	8.08	−1.71	0.09	0.22

## Discussion

Although the only statistically significant change was found on the WHO-5, there were small positive improvements on all the measures used. Therefore, it was felt that there was some basis for rolling out the Hummingbird Project to a larger cohort of secondary school children. However, before this took place, the PPI was revised to improve its efficacy. The session on mental illness and stigma was removed as these subjects were not consistent with a PPI. This session was replaced with a short discussion with participants regarding stigma, taking place in session 1. It was felt that this would allow students to raise any questions or concerns they might have regarding mental illness. The decision was taken to shorten the length of the PPI as many half-terms in the standard academic calendar are shorter than 8 weeks. Therefore, the intervention might be interrupted by a break of a week or more. Having the sessions take place on consecutive weeks would keep the ideas fresh in students’ minds and allow them to consider them together. It was also decided that the GRIT scale should be removed from the evaluation questionnaires. The GRIT scale was removed for three reasons. Firstly, the scores on this scale showed very little change. Secondly, the GRIT scale and the BUSS scale measure similar constructs. Thirdly, it was felt that reducing the length of the questionnaires would compensate for the time lost in reducing the length of the PPI in order to better teach students about the concepts in question.

The main study involved altering the PPI with consideration of feedback from parents, teachers, students, and researchers, as well as results from the scales used, to improve its efficacy. The intervention was then rolled out to a larger number of schools in the local area and assessed using similar scales along with open-ended questioning of students and teachers.

## Materials and Methods

The revised Hummingbird Project was shortened to involve 6 weekly 1-h sessions, taking place during normal timetabled hours, delivered by a single researcher, with support from undergraduate and MSc Psychology students. Session 1 involved an introduction to the PPI for students and teachers and a discussion of stigma around mental illness. Sessions 2–5 covered concepts from Positive Psychology. These sessions took place with students, with the sessions covering happiness, resilience and character strengths, hope, growth mindsets and gratitude, and mindfulness, respectively. The final session included an overview of the topics covered and advice for students on how to build on the concepts for the future, as in the pilot.

### Participants

Participants in the main study were an opportunity sample of 1,054 students at 14 schools in the North West of England. Participants were recruited by emailing the Head Teachers and SENCOs of several schools in the region. Inclusion criteria were studying at a secondary school and willingness to take part in the study. Students were selected by their respective schools, without researcher input, using a range of criteria. These included; form group, Personal, Social and Health Education (PSHE) group, timetabled lesson group, or perceived need for intervention. Participant ages ranged from 11 to 18 and 57% were female. Each student received a workbook in which they could record all of the work they did in sessions and at home, as well as any thoughts and feelings they had about the experience. They were also given questionnaires as outcome measures.

### Instruments

Questionnaires contained three standardized quantitative measures. These were the same battery of scales used in the pilot project, minus the GRIT scale.

### Data Collection Procedures

Outcome measures were administered to participants at the start of session 1 and the end of session 6 of the PPI. As in the pilot study, participants were asked to be as honest as possible and informed of their right to withdraw from the study. They were informed that school grades would not be affected by participation in the study or by their answers to the questions. Upon completion of pre- and post-intervention questionnaires, questionnaires were coded as stated above, with negatively coded items being reverse coded. Only the BUSS contained negatively coded items.

### Data Analysis Procedures

Data were analyzed using SPSS (version 25). Descriptive statistics were generated and reliability analyses were conducted for all scales. As parametric tests have been demonstrated to be valid for large sample sizes due to the central limit theorem (Lumley et al., 2002), paired-samples *t*-tests were conducted to compare scores on the three scales before and after participation in the PPI and to analyze the impact of the PPI. A one-way MANOVA was also performed in order to investigate whether student selection type had an impact on the PPI’s impact on outcome measures.

## Results

Paired sample *t*-tests were performed. Table 2 shows descriptive statistics from these tests. Results from the Hummingbird Project main study show that the PPI resulted in a positive change in all of the outcome measures in pre- and post-intervention testing, showing the average child has seen an improvement in their well-being (WHO-5), an improvement in their level of resilience (BUSS), and an increased level of hope for the future (CHS).

**TABLE 2:** Descriptive statistics for main study.

Scale	n	Start mean	*SD*	End mean	*SD*	*t*	*p*	Cohen’s d
WHO-5	657	12.47	5.43	12.94	5.95	−2.46	0.01	0.1
BUSS	584	36.63	7.03	37.23	6.44	−2.84	0.01	0.12
CHS	647	20.32	6.76	21.64	6.89	−6.11	<0.01	0.24

It was then decided that it might be useful to investigate whether student selection type had an impact on the PPI’s impact on outcome measures. A one-way MANOVA was performed. Table 3 shows descriptive statistics for this MANOVA. A significant multivariate main effect was found for well-being (WHO-5), *F*(1, 652) = 6.748, *p* = 0.010. The same MANOVA revealed a significant multivariate main effect for resilience (BUSS), *F*(1, 579) = 8.73, *p* < 0.01. There was also a significant multivariate main effect for hope (CHS), *F*(1, 643) = 38.50, *p* < 0.01. These results show that the well-being, resilience, and hope, of students selected based on perceived need were all improved significantly more by the Hummingbird Project than those of the students who took part in the project in groups that they would ordinarily be in during a normal school day.

**TABLE 3:** Descriptive statistics for main study by student selection type.

	Scale	n	Start mean	*SD*	End mean	*SD*	*t*	*p*	Cohen’s d
Non-selected	WHO-5	338	13.42	5.15	13.68	5.73	−0.99	0.32	0.11
	BUSS	312	38.11	6.70	38.61	6.47	−1.80	0.07	0.20
	CHS	344	21.78	6.30	22.85	6.45	−3.85	<0.01	0.42
Selected	WHO-5	301	11.42	5.54	12.16	6.08	−2.60	0.01	0.30
	BUSS	269	35.04	7.10	35.81	6.08	−2.31	0.02	0.28
	CHS	301	18.70	6.88	20.33	7.09	−4.80	<0.01	0.55

## Discussion

Results shown in the main study of the Hummingbird Project are promising, with positive improvements on all outcome measures. Results show small effect sizes across all of these factors, with the largest effect size being 0.24. Over time, the effects found here might increase. Once participants have had time to reflect on the knowledge they have gained during the course of the PPI and practice the skills and techniques that they have learned in the 6 weeks of sessions, they may become more skilled in attending to their own mental health. Follow-up testing would be needed to test this. Budget and time constraints meant this was not possible during year 1 of the main study of the Hummingbird Project. Later phases of the project may provide time and money, and some of the schools involved in the PPI have expressed openness to including follow-up testing. A meta-analysis of meta-analyses conducted by Tanner-Smith et al. (2018) found universal prevention programs of this type, delivered to school-aged children, have effect sizes within the range of 0.07–0.16. Therefore, the results found in the current study show that the Hummingbird Project has a comparatively large effect on the mental health of participants. Given that school-based preventative interventions in the UK have been shown to have a cost-benefit ratio of at least 1:10 and potentially more than 1:80 (Arango et al., 2018), the Hummingbird Project might potentially have a large effect on the mental health of school-aged children, at a very encouraging cost-benefit ratio. The project was also delivered by a single member of staff without specialist doctoral training, with the support of current MSc and BSc level students, demonstrating such interventions do not require large amounts of previous training. As noted earlier Farnfield and Kaszap (1998), stated the factors that young people look for in those who intervene in their mental health are their personal qualities not the profession of the trainer. Also, considering the findings regarding the high levels of teacher stress (Skaalvik and Skaalvik, 2015) and the lack of teacher training in mental health (Byrne et al., 2015) previously mentioned, having an intervention that is easily delivered by staff with minimal training has the potential to have a positive effect on staff well-being (or at least mitigate the negative effect that other, more labor-intensive interventions might cause) as well as improve student mental health.

A number of issues arose during the delivery of the Hummingbird Project. One such issue was that low budgets led to shortages in time, space, and resources in schools across the country. These shortages can have a negative impact on the ability to effectively deliver a PPI in those schools. Hence a tolerance has been built into this project, allowing individual sessions to be shorter or longer than originally intended. In this way, should an unexpected problem arise, a particular activity can be altered, or an alternative activity might be introduced. One problem that was encountered when delivering the PPI in schools in the half-term before Christmas was that schools had seasonal events, exams, and double-booked rooms, leading to disruptions and missed sessions. An unforeseen issue was that students are used to questions being asked in the school setting having well-defined, right and wrong answers. Students were reluctant to answer questions about their own experiences and opinions, not only due to concerns about revealing personal information to peers and staff but also because they were concerned they might give the “wrong” answer. To overcome this reluctance, at all opportune moments, the anonymity and privacy afforded by the PPI were highlighted, as well as the subjective nature of happiness, and the fact that the PPI sessions are a safe place to discuss thoughts and feelings. Another issue that arose whilst delivering the PPI was that schools were inclined to assign students to the project according to perceived need for some kind of intervention into their mental health. This may be due to the reductions in school and CAMHS’ budgets discussed previously. These budget reductions have led to insufficient mental health interventions. Therefore, schools might be more likely to assign students with perceived mental health problems to an intervention such as the Hummingbird Project as these children are not receiving the care they require. However, this is problematic in the case of the Hummingbird Project. The project has been designed to be a universal intervention for all students. It is not intended to target children with severe mental health problems and therefore it may not be appropriate for these children. Previous research shows that when students are targeted for intervention based on perceived need, they tend to respond poorly to the intervention (Salerno, 2016) as they feel that they are being stigmatized and therefore are more likely to act out or even fail to complete the intervention. Close to half (509) of all the students who took part in the current study were selected by their schools based on a perceived need for mental health intervention. However, the results above show that these students actually showed larger increases in the scores on all measures than the students who were in groups they would usually be in during a normal school day. These results are encouraging as it shows that the project can potentially be used to improve the mental health of school-aged children with existing mental health problems. These results also show that teachers know their students well and are well-placed to identify students who require intervention into their mental health, since the pre-intervention scores of the selected students were significantly lower on all measures than those of the students who were in their usual groups. This was also true at post-test. Selected students showed larger improvements in their scores than non-selected students. The improvements in scores of the selected students were all statistically significant, whereas those of the non-selected students only showed significant improvements in their levels of hope (CHS). However, the results found here might be the result of a ceiling effect that limits the ability of the PPI to improve the mental health of those students who are already flourishing.

The Hummingbird Project compares quite favorably to the limited number of PPIs that have been delivered in school settings. A much smaller study of a multi-component PPI in schools showed similarly positive outcomes (Freire et al., 2018). The team conducting that study encountered similar difficulties to those experienced in the current study regarding randomization. However, the current PPI was delivered to a far higher number of participants and was delivered by current university students, showing it is readily reproducible. A previous study involved the delivery of a PPI as a year-round addition to the timetable (Shoshani and Steinmetz, 2014). The Hummingbird Project is much shorter, taking at most 6 weeks to complete, and therefore does not require the integration of PP into teacher training, school leadership training, or into the wider educational culture. The current PPI has also been delivered with relatively low staff numbers and without the need for intensive staff training, therefore comes at a far lower cost. One PPI involved parents in PPI sessions, thus introducing more difficulties regarding recruitment (Roth et al., 2017). This PPI only targeted small groups of students whereas the Hummingbird Project could be delivered to entire year groups. Delivering to year groups also helps overcome difficulties around psychological interventions regarding stigma. PPIs targeting at-risk students have been shown to be effective (McCarty et al., 2011; Muro et al., 2018). The current study shows that selected students benefit the most from a universal PPI. Delivering universal PPIs to whole year groups helps reduce stigma by not singling out at-risk students, whilst also improving the well-being of students who are not at-risk. The current PPI also compares favorably to those which focus on a single aspect of PP (Froh et al., 2009; Quinlan et al., 2015; Sapthiang et al., 2019). The Hummingbird Project gives students an opportunity to sample a range of PP concepts and activities, enabling them to choose the activities that they feel suit them best. This improves person-activity fit, encourages better engagement, and has the potential to improve outcomes (Schueller and Parks, 2014). Person-activity fit might also explain previous results regarding mixed outcomes for vanguard PPIs such as the Penn Resiliency Program (Bastounis et al., 2016) which focus on a single aspect of PP, potentially reducing broad appeal.

One of the limitations of the Hummingbird Project is that it was coordinated and delivered by a single member of staff, supported in sessions by volunteers. For the PPI to have a sustainable future, it will need to be scaled up so that it can be delivered by multiple trainers on multiple sites. Yet fidelity to original course materials and procedures is a significant predicting factor in outcomes of universal school-based PPIs (Mackenzie and Williams, 2018). If the Hummingbird Project is adapted to become a train-the-trainer model it will be vital to take steps to ensure that the trainers who end up delivering to children and adolescents are monitored regularly and supported in their endeavors. Issues regarding fidelity might be overcome by offering an online version of the Hummingbird Project. Using online materials would ensure that children and adolescents accessing the PPI are working from the same resources. An online version of the PPI might also better appeal to children and adolescents, as “digital natives.” Using an online version of a PPI comes with its own difficulties (Neumann, 2016). It may be necessary to ensure that a human aspect to the PPI is maintained so that children and adolescents can be offered guidance from a skilled member of staff who can answer any questions or concerns they might have. Having such a staff member will help children and adolescents use the resources correctly, reassure them, and make sure their needs are being met. A second limitation of the Hummingbird Project is that the PPI is designed to be delivered to high school students. It has been shown that the prevalence of mental health problems in primary school students is at 7.7% for all students and as high as 10.2% for boys (Green et al., 2005). It would therefore be advisable to attempt to work with students before they reach high school. However, it will be necessary to rewrite the PPI so that the contents are more readily understood by a primary school-aged cohort. Another potential change to the Hummingbird Project is to adjust the order of sessions such that the mindfulness session takes place earlier on in the process. Having learned about and practiced mindfulness in an earlier session, the students will be better able to focus when learning some of the other, less familiar concepts (Hyland, 2011). Changing the order of sessions might present an opportunity to better link the concepts so that students find them more readily understandable by making the content of each session lead on to the next and giving the project more of a coherent narrative. Another change is to communicate in stronger terms to schools involved that it is intended as a universal intervention for all students. This has the potential to increase the number of students who can participate. It could also reduce the negative effect of students feeling that they are being singled out for participation in the project and therefore increasing perceived stigmatization (Chandra and Minkovitz, 2006). Future research should also attempt to establish whether or not the PPI improves negative outcomes for participants by including measures of depression and anxiety. Mental health problems are the leading contributors to the health burden in children and adolescents (Stockings et al., 2016). Showing a decrease in levels of depression and anxiety as a result of participation in the Hummingbird Project would represent a significant step in the intervention in child and adolescent mental health, as the evidence for the efficacy of other PPIs is mixed (Bolier et al., 2013).

### Implications

The current study demonstrates that school-based PPIs do not necessarily require large amounts of staff training in order to be efficacious and therefore could have positive effects on teachers as well as students. However, the next step in this journey involves providing evidence for the efficacy of this PPI in reducing mental ill-health in this cohort. Another important step in improving the mental health of the region’s children and adolescents is to establish the sustainability of PPIs delivered in such a way, without compromising the aspects that have a positive impact. This might necessitate an approach that involves pairing a train-the-trainer model with online resources for teachers, students, parents, and carers. The findings of Green et al. (2005) discussed above, also make it clear that intervention into child mental health must begin at an earlier age. Therefore, it will be necessary to adapt the approach taken in the current study to a primary school-aged population.

## Conclusion

The current study, and the Hummingbird Project itself, represent a significant step forward in the intervention into the mental health of secondary school-aged students in the North West of England. The project has led to improvements in the flourishing of over 1,000 secondary school-aged children in the region. The current study shows that the Hummingbird Project occupies a happy medium in the dichotomy between mental health professional-delivered, prescriptive interventions and the kind of interactive, self-management encouraging interventions that are preferred by older adolescents (Brown et al., 2019). As stated by one of the students that took part in the PPI, “The Hummingbird Project is amazing! Very supportive and helpful. Highly recommended.” This study provides evidence that even a short 6-week Multi-component PPI, delivered by staff without extensive training, can lead to significant improvements in well-being, hope and resilience in high school students.

## Data Availability Statement

The datasets generated for this study are available on request to the corresponding author.

## Ethics Statement

The studies involving human participants were reviewed and approved by the University of Bolton Ethics Committee. Written informed consent to participate in this study was provided by the participants’ legal guardian/next of kin.

## Author Contributions

IP was the main author and lead researcher. CK supervised and assisted in the conception of the research in phases 1–3, and contributed to the writing-up of these phases. JC supervised and guided the research in phase 4 and edited the final manuscript. MT provided assistance in phase 4 of the research, conducted statistical analysis of results, and contributed to interpretation of the final conclusions. All authors contributed to the article and approved the submitted version.

## Conflict of Interest

The authors declare that the research was conducted in the absence of any commercial or financial relationships that could be construed as a potential conflict of interest.
